# Identifying and Predicting Autism Spectrum Disorder Based on Multi-Site Structural MRI With Machine Learning

**DOI:** 10.3389/fnhum.2021.765517

**Published:** 2022-02-22

**Authors:** YuMei Duan, WeiDong Zhao, Cheng Luo, XiaoJu Liu, Hong Jiang, YiQian Tang, Chang Liu, DeZhong Yao

**Affiliations:** ^1^Department of Computer and Software, Chengdu Jincheng College, Chengdu, China; ^2^College of Computer, Chengdu University, Chengdu, China; ^3^The Key Laboratory for Neuro Information of Ministry of Education, Center for Information in Bio Medicine, High-Field Magnetic Resonance Brain Imaging Key Laboratory of Sichuan Province, School of Life Science and Technology, University of Electronic Science and Technology of China, Chengdu, China; ^4^Department of Abdominal Oncology, Cancer Center, West China Hospital, Sichuan University, Chengdu, China; ^5^Department of Neurosurgery, Rui-Jin Hospital, Shanghai Jiao Tong University School of Medicine, Shanghai, China

**Keywords:** autism spectrum disorder, structural MRI, multi-site data, machine learning, searchlight technique

## Abstract

Although emerging evidence has implicated structural/functional abnormalities of patients with Autism Spectrum Disorder(ASD), definitive neuroimaging markers remain obscured due to inconsistent or incompatible findings, especially for structural imaging. Furthermore, brain differences defined by statistical analysis are difficult to implement individual prediction. The present study has employed the machine learning techniques under the unified framework in neuroimaging to identify the neuroimaging markers of patients with ASD and distinguish them from typically developing controls(TDC). To enhance the interpretability of the machine learning model, the study has processed three levels of assessments including model-level assessment, feature-level assessment, and biology-level assessment. According to these three levels assessment, the study has identified neuroimaging markers of ASD including the opercular part of bilateral inferior frontal gyrus, the orbital part of right inferior frontal gyrus, right rolandic operculum, right olfactory cortex, right gyrus rectus, right insula, left inferior parietal gyrus, bilateral supramarginal gyrus, bilateral angular gyrus, bilateral superior temporal gyrus, bilateral middle temporal gyrus, and left inferior temporal gyrus. In addition, negative correlations between the communication skill score in the Autism Diagnostic Observation Schedule (ADOS_G) and regional gray matter (GM) volume in the gyrus rectus, left middle temporal gyrus, and inferior temporal gyrus have been detected. A significant negative correlation has been found between the communication skill score in ADOS_G and the orbital part of the left inferior frontal gyrus. A negative correlation between verbal skill score and right angular gyrus and a significant negative correlation between non-verbal communication skill and right angular gyrus have been found. These findings in the study have suggested the GM alteration of ASD and correlated with the clinical severity of ASD disease symptoms. The interpretable machine learning framework gives sight to the pathophysiological mechanism of ASD but can also be extended to other diseases.

## 1. Introduction

Autism Spectrum Disorder, known as ASD, is a complex neuro-developmental disorder and has been characterized by a series of symptoms including early-onset difficulties in social communication as well as restricted, repetitive behaviors and interests (Pagnozzi et al., 2018). The symptoms of ASD generally occur within the first 3 years of life and tend to last even one's whole life (Hazlett et al., 2017). ASD brings significant impairments on an individual's language, emotions, behavior, self-control, learning, and memory and also is accompanied by intellectual disability. Moreover, it is reported that patients with ASD are far more likely to encounter premature death than healthy controls (Hirvikoski et al., 2015). According to the Morbidity and Mortality Weekly Report (MMWR) Series published by the Centers for Disease Controls and Prevention (CDC) in the United States, the prevalence of ASD among children has increased from 1 in 150 to 1 in 54 over 16 years (from 2000 to 2016) and the incidence rate of ASD was 4.3 times higher in boys than girls (Maenner et al., 2020) in 2016. For each patient with ASD, the average lifetime social cost is approximately $3.6 million (Cakir et al., 2020).

Actually, if ASD is unable to be detected and intervened at an earlier age, the impairments are irreversible. Therefore, early and accurate identification and diagnosis are crucial to improving the life quality of ASD patients and their families. Unfortunately, it is notoriously difficult to diagnose, especially in children, since the cause of ASD is a result of combined factors, including genetics, the structure and function of the brain, as well as environmental influences (Rakić et al., 2020). Until now, there are still no effective medical treatments for ASD. For the current practice guidelines to assess, diagnose and treat ASD, it is recommended to use the behavioral observation of symptomology following the Diagnostic and Statistical Manual (Fifth Edition) (DSM-5) (American Psychiatric Association, 2013) symptom criteria and the International Classification of Mental and Behavioral Disorders (Tenth Edition) (ICD-10) (Organization, 1993). However, uniformity is lacking while using these practice guidelines, so it is probably prone to misdiagnosis (Eslami et al., 2021). Furthermore, these guidelines cannot point out the biological bases related to behavioral symptoms due to unclear neuroanatomy. Finally, these limitations have resulted in calls for more optimal diagnostic approaches for ASD.

In the last few decades, advances in non-invasive neuroimaging techniques and analysis have provided crucial knowledge to uncover patterns of brain structure and function that would be symptomatic for the autism spectrum. The vast majority of statistical methods on Structural MRI have intended to explore the common patterns between patients with ASD and healthy groups, but previous volumetric and morphological analysis on structural MRI often has derived contradicted results. For example, some research work reported decreased volumes of the amygdala for ASD (van Rooij Daan et al., 2017) while others did not find significant alterations (Maier et al., 2015). Focusing on the hippocampus volumes, some reported its reduction, others reported its enlargement or no changes (Barnea-Goraly et al., 2014; Maier et al., 2015). Xiao et al. (2014) has found that both gray and white matter (WM) has a significant increment with ASD, and Hazlett et al. (2017) has pointed out brain volume overgrowth is related to the emergence and severity of ASD. While Palmen et al. (2005) and Jou et al. (2011) have noted that there is no difference or decreased WM volume between ASD and healthy controls, and Riddle et al. (2016) conducted voxel-based morphometry analysis and found that the total brain volume and the left anterior superior temporal gyrus increased for children aged 2–4 with ASD. But these brain structural abnormalities are subtle at later ages (Riedel et al., 2014). These inconsistent findings are most likely due to different collecting approaches and limited sample size with heterogeneous characteristics of subjects (Riddle et al., 2016). Moreover, traditional statistical analysis is based on mass univariate techniques which process a single voxel independently and ignore the relationship between voxels (Bonnici et al., 2012; Samartsidis et al., 2016). Furthermore, it defines the common pattern at the level of groups and is unable to predict the unknown sample at the level of individuals (Zhutovsky et al., 2019; Hu et al., 2021).

Most recently, the rapid advance of machine learning has made it becomes possible to explore the underlying neural mechanisms and provide accurate predictions and convincing explanations for ASD from various aspects (Khodatars et al., 2020; Eslami et al., 2021). Knutson (2013) has pointed out that machine learning can detect differences in neuroimaging data that might not be detected with traditional univariate analysis. In previous studies, typical statistical machine learning and deep learning have been utilized to identify ASD from NC in terms of structural and functional alterations. Statistical machine learning requires the design of handmade features (feature extraction/feature selection) and implement the identification of patients with ASD based on these features (feature classification). Ecker et al. (2010) has applied SVM to investigate the whole-brain differences of GM and WM volume on 44 subjects and obtained significant predictive power. Additionally, it has been found that these brain differences are related to symptom severity. Ecker et al. (2010) and Wee et al. (2014) have extracted morphological features based on structural images and used SVM or multi-kernel technique to achieve satisfactory results. Furthermore, Zheng et al. (2018) have constructed a multi-feature-based network based on morphological features to explore the cortico-cortical similarities of ASD. Bilgen et al. (2020) have modeled the morphological relationship between pairs of ROIs with a cortical networks and verified the classification performance of different machine learning methods. Concerning female children, Calderoni et al. (2012) have detected the abnormality of the gray matter volume based on SVM-RFE (Leung et al., 2006; ChenZhiHong et al., 2020) and found the increased cortical volume in some brain regions involving the left superior frontal gyrus (SFG). In addition, bilateral SFG and right temporoparietal junction (TPJ) resulted in the appearance of some atypical symptoms of ASD and might be relevant to the pathophysiology of female children in ASD. These findings are helpful to reveal the important influence of the structural alterations and the relationship between the brain structure and the pathophysiology of ASD.

However, the lack of a sufficiently large sample at a single site probably leads to poor generalizability that is notably serious for neuroimaging due to limited participants and super-high dimensionality of data. Consequently, the investigation of large sample data from multi-site has attracted increasing attention. Some studies (Spera et al., 2019; Mwiza et al., 2020) have figured out the superiority of machine learning for the classification of multi-site data based on fMRI or the combining structural and MRI in the Autism Imaging Data Exchange database (Di Martino et al., 2017). Due to the excellent performance of deep learning in the field of artificial intelligence on large sample data, some researchers have begun to detect abnormalities of functional connectivity based on deep learning. Deep learning has combined dimensionality reduction and feature classification and implemented the end-to-end classification model automatically, which has achieved satisfaction performance (Eslami et al., 2019; Sherkatghanad et al., 2020). Furthermore, some attempts have been done to fuse structural and functional features with the model of deep learning to improve the classification performance (Rakić et al., 2020). But it cannot be denied that deep learning handles data with the mechanism of a black box and it is so hard to identify the abnormal brain regions and connect the classification accuracy with the underlying mechanism of ASD. Furthermore, multi-site data also has brought the issue of data heterogeneity due to different scanning parameters and participant populations. The direct way to address the heterogeneity issue is to apply dimensionality reduction to transform source data into features in the field of machine learning (Wang et al., 2020). Furthermore, these studies also utilized leave-one-site-out cross-validation to evaluate the classification performance in the expectation of reducing the impact of heterogeneity simultaneously (Rakić et al., 2020; Eslami et al., 2021). In order to make the results robust, Ashourvan et al. (2016) have further proposed intra-site cross-validation and inter-site cross-validation and achieved 65% accuracy with functional connections (FC) to identify ASD from normal control.

In fact, detecting the structural/functional brain alterations is vital to reveal the pathological mechanism of ASD. In particular, these brain regions with obvious differences can be recognized as the neuro-imaging biomarkers related to the disease. Based on this kind of neuro-imaging biomarkers (brain regions), achieving excellent classification performance even from different multi-sites would be the most desirable and helpful in the clinical diagnosis. Meanwhile, aiming at a few investigations on volumetric changes based on machine learning, this study has applied machine learning techniques followed the unified framework to implement model-level, feature-level, and biology-level assessment successively. First of all, a searchlight-based classification method has been used to detect the volumetric changes locally and some candidate brain regions have been defined based on the areas of the volumetric changes at the model-level assessment; Regarding distinguished brain regions, this study has processed the “visual lesion” analysis at the feature-level assessment. Stability based on nested cross-validation and multi-site validation of each region has been evaluated. The candidate regions with good stability performance have been preserved and considered as candidate biomarkers related to ASD. Finally, this study investigated the relationship between candidate biomarkers and symptom severity and analyzed our results with previous findings.

The main contributions of the study are discussed as follows: (1) Previous machine learning studies on ASD mainly focus on the classification performance or the important features. Furthermore, this study paid attention to the interpretability of the machine learning model to explore abnormal brain regions related to ASD and conducted model level and feature level assessment to ensure the robustness and stability of the results. (2) The correlation analysis between abnormal brain regions and clinical severity in our study has further proved the relationship between the volume changes of some specific brain regions and the clinical symptom. (3) The findings in our study are partly consistent with previous research work. The abnormal gray matter (GM) volume in the temporal lobe, Broca and Wernicke area probably provides the support for the social brain hypothesis and the broken mirror theory of ASD, which is helpful to understand the neuroanatomy of ASD.

The structure of this study is as follows: First, in section 2, we provide a brief introduction to the pre-processing procedure and statistical analysis of sMRI data. In section 3, we describe the machine learning workflow in detail. Experimental results and discussion are provided in section 4. Finally, in section 4, we conclude the study and discuss the future direction.

## 2. Material

### 2.1. Participants

All data carried in the present study came from the Autism Imaging Data Exchange (ABIDE II) (http://fcon_1000.projects.nitrc.org/indi/abide/abide_II.html). Briefly, ABIDE with ABIDE I and ABIDE II is a public repository that provides structural MRI and resting-state fMRI acquired on ASD and matched control subjects for the purpose of data sharing and scientific research (Martino et al., 2013). The ABIDE II includes 1,114 data sets from 19 independent sites which comprise 521 participants with ASD and 593 typically developing controls (TDC) with the age from 5 to 64. All participants in ABIDE have received approval from the Institutional Review Board (IRB) of each site. In the present study, we have selected three independent datasets from Georgetown University (GU), Oregon Health and Science University (OHSU), and University of California Los Angeles (UCLA) which are collected by the same scanner (Siemens) and all participants are children with the age from 7 to 15 to reduce the variability of multi-site neuroimaging data. Since GU has the greatest participants, machine learning methods were conducted on GU with nested cross-validation. Furthermore, we also trained machine learning models for GU and tested them on OHSU and UCLA to verify their robustness. Demographics information of participants is summarized in Table 1. The scanning parameters of the three sites are listed in Table 2.

**Table 1:** Demographics information.

**Site**	**Gender(M/F)**		**Age**		**Full scale IQ**	
	**NC**	**ASD**	**NC**	**ASD**	**NC**	**ASD**
GU	28/27	43/8	10.448 ± 1.696	10.896 ± 1.535	121.46 ± 13.808	118.3 ± 15.377
OHSU	27/29	30/7	10.38 ± 1.636	11.81 ± 2.271	117.46 ± 11.968	105.97 ± 16.734
UCLA	11/5	15/1	9.3 ± 2.09	11.13 ± 2.247	115 ± 13.05	102.06 ± 13.959

**Table 2:** The scanning parameters of structural MRI imaging in Georgetown University (GU), Oregon Health and Science University (OHSU), and University of California Los Angeles (UCLA) with Siemens.

**Site**	**Voxel size(mm^3^)**	**Flip angle(deg)**	**FOV(mm)**	**TR(ms)**	**TE(ms)**	**T1(ms)**
GU	1 × 1 × 1	7	256 × 256	2530	3.5	1100
OHSU	1 × 1 v 1.1	10	256 × 256	2300	3.58	900
UCLA	1 × 1 × 1.2	9	256 × 256	2300	2.86	853

### 2.2. MRI Data Pre-processing

All structural images were processed using the SPM8 package (Welcome Trust Center for Neuroimaging, London, UK, http://www.filion.ucl.ac.uk/spm/software/spm8/) and the VBM8 (Voxel-Based Morphometry) toolbox (http://dbm.neuro.uni-jena.de/vbm) running under Matlab R2014a (Mathworks). At first, all T1-weighted images were corrected for bias-field inhomogeneities and then segmented into GM, WM, and CSF (cerebrospinal fluid) based on a tissue probability map (Mazziotta et al., 1995). The segmented GM/WM image was spatially normalized to the “IXI500_MNI152” template based on the DARTEL algorithm (Ashburner, 2007). After that, non-linear warping for the effect of spatial normalization was corrected to generate GM/WM modulated normalized images. Finally, spatial smoothing (Gaussian kernel with 6 mm full-width at half-maximum) was conducted on GM/WM images to remove noise.

### 2.3. Statistical Analysis

In the present study, a two-sample *t*-test has been employed on the GU dataset with age, gender, Total Intracranial Volume (TIV) as the effect-of-no-interest covariates to identify group differences between ASD and TDC. A significance level of *p* < 0.001 (uncorrected) was established with an extent threshold of 50 voxels. Meanwhile, an absolute threshold mask of 0.1 was used on GM/WM volume images to avoid potential edge effects.

## 3. Methods

This study aimed to identify the brain abnormality and predict ASD from TDC *via* machine learning techniques. However, neuroimaging-based ML models like the “black-box” and unable to be understood from the prospect of neuroscience. To address this issue, Kohoutov et al. (2020) has developed a unified framework to enhance the interpretability of ML models and provide mechanistic insights into underlying neural or disease processes. The proposed framework contains a three-stage process of assessment including Model-level assessment, Feature-level assessment, and Biology-level assessment. In the first stage, the ML model has been built from observations and assessed in terms of its sensitivity, specificity, and generalizability. In the second stage, significant features have been identified from a prediction within the model. Finally, the neuroscientific plausibility of the ML model has been proved with evidence from previous literature and other studies.

**Figure 1:**
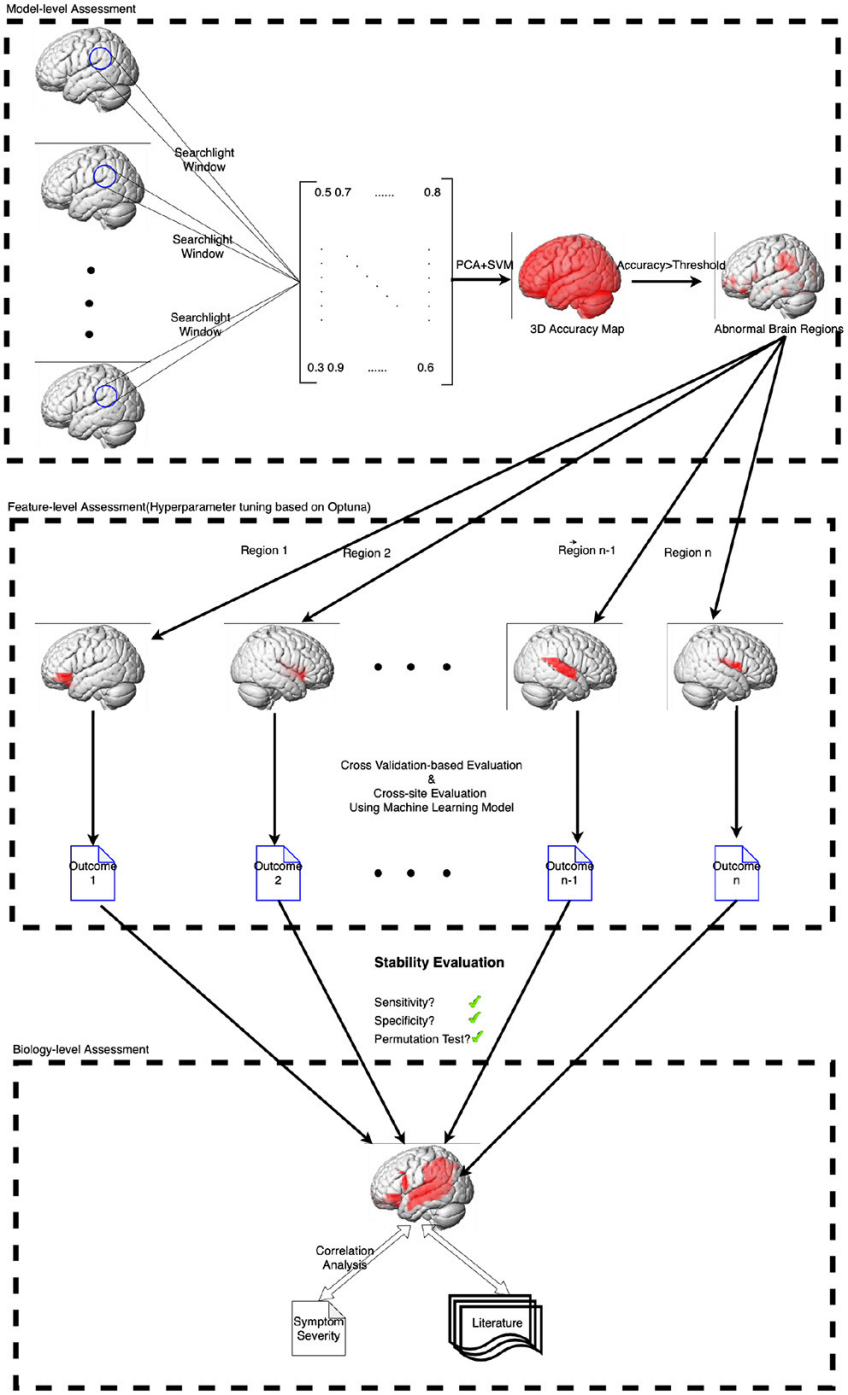
The machine learning workflow proposed in the study.

However, ML models based on neuroimaging are often built on numerous features and limited participants, which makes the model is prone to overfitting and leads to poor generalization and expensive computational cost even if dimensional reduction techniques have been used. Moreover, isolated features are often insufficient to acquire satisfactory predictive performance and explain the model performance. Consequently, the study has designed a neighborhood-to-regional machine learning workflow within this unified framework to identify structural alteration and discriminant ASD from TDC. The workflow proposed in the study has been illustrated in Figure 1.

### 3.1. Model-Level Assessment

First, the study has built an ML model based on the searchlight technique (Kriegeskorte et al., 2006). A spherical window is centered at each voxel to generate a data matrix from the voxel and its neighbors. In light of the spherical window, PCA(Principal Component Analysis) has been used to reduce the dimensionality of the matrix, and SVM(Support Vector Machine) has been used to achieve the classification.

#### 3.1.1. Principal Component Analysis

Supposed data matrix obtained from training data $x=\left\{x_{1},x_{2},...x_{m}\right\}\in {\mathbb{R}}^{m\times n}$ is obtained from a spherical window, where *m* is the number of subjects in the training dataset, *n* represents the voxel number centered a specific voxel within a spherical window, PCA (Wold et al., 1987) has been used to reduce the dimensionality of the matrix by transforming high-dimensional data into lower-dimensional features while preserving its maximum variance. To this end, data points are projected from high-dimensional space to low-dimensional space with the following linear combinations:


(1) $$\begin{array}{l} \text{y}=\overset{n}{\underset{j=1}{\sum }}a_{j}{\text{x}}_{j}=Xa \end{array}$$

where $a=\left\{a_{1},a_{2},...,a_{n}\right\}\in {\mathbb{R}}^{n\times k}$ and k ≪ n, **y** is the low-dimensional features. Meanwhile, the variance of the low-dimensional feature is given by:


(2) $$\begin{array}{l} \mathrm{var}\left(\text{X}\text{a}\right)={\text{a}}^{T}\text{S}\text{a} \end{array}$$

where **S** is the symmetric covariance matrix of data samples. Hence, the linear combination with maximum variance can be achieved by optimizing the following problem:


(3) $$\begin{array}{l} \max{\text{a}}^{T}\text{S}\text{a}s.t.{\text{a}}^{T}\text{a}=1 \end{array}$$

In light of the Lagrangian multiplier method with the restrictions of orthogonality of different coefficient vectors, we can obtain the following equation easily:


(4) Sa−λa=0⇔Sa=λa

where ***a*** is the orthonormal eigenvector of the covariance matrix **S** and λ refers to the corresponding eigenvalue. Thus, the maximum variance corresponds to the largest eigenvalue as follow:


(5) $$\begin{array}{l} \max\mathrm{var}\left(\text{X}\text{a}\right)=\max{\text{a}}^{\text{T}}\text{S}\text{a}=\max\lambda {\text{a}}^{\text{T}}\text{a}=\max\lambda \end{array}$$

As a consequence, the eigenvectors of **S** corresponding to the first **k** largest eigenvalues can be considered as the coefficient vectors ***a*** and these linear combinations **Xa**_*k*_ are called the principal components (PCs) of the dataset. The quality of a given PC **Xa**_*j*_ is measured according to the following proportion of total variance:


(6) $$\pi_{j}=\frac{\lambda_{j}}{\sum_{j=1}^{n}\lambda_{j}}=\frac{\lambda_{j}}{\mathrm{tr}(S)}$$

where tr(**S**) denotes the trace of **S**, and λ_*j*_ is the *j*th eigenvalue of **S**. The proportion of total variance preserved by a set of **S** of PCs can be expressed as a percentage of total variance as follows:


(7) $$\sum_{j=1}^{k}\pi_{j}=\frac{\sum_{j=1}^{k}\lambda_{j}}{\mathrm{tr}(S)}$$

In practice, it is common to use some predefined percentage of the total variance to decide how many PCs should be retained, rather than setting the number of the coefficient vector **k** directly. In our study, 80% of total variability has been used.

#### 3.1.2. Support Vector Machine

After that, supposed a set of feature-label pairs(*f*_*i*_, *y*_*i*_), $i=1,\ldots ,m,\;f_{i}\in {\mathbb{R}}^{k},y_{i}\in \left\{-1,+1\right\}$, the classification with linear SVM (Fan et al., 2008) has been implemented according to solving the following unconstrained optimization problem:


(8) $$\begin{array}{l} \underset{w}{\min}\frac{1}{2}w^{T}w+C\overset{l}{\underset{i=1}{\sum }}\xi \left(w;f_{i},y_{i}\right) \end{array}$$

where *C* is a penalty parameter and the loss function $\xi \left(w;f_{i},y_{i}\right)=\max{\left(1-y_{i}w^{T}f_{i},0\right)}^{2}$.

When a new testing data point *x* arrives, it can be projected into low-dimensional space by PCA as follows:


(9) $$\begin{array}{l} {\text{x}}^{\prime }=\text{x}\text{a} \end{array}$$

and then the low-dimensional feature **x**′ is predicted as positive if ***w***^*T*^***x*** > 0 and negative, otherwise. In the present study, the number of the coefficient vector **k** of PCA is determined when preserving the energy of PCs is 80% and the penalty parameter *C* = 1 of linear SVM is used in default.

The classification accuracy of a spherical window around a specific voxel has indicated how well centered voxel in the local spherical neighborhood differentiates between different groups. According to slide the spherical searchlight window on each voxel of GM/WM images, a 3D accuracy map has been obtained to explore the local spatial pattern of GM/WM volume. The ML model based on the neighborhood window is useful to relieve overfitting and computational cost problem.

In order to assess the robustness of the results, the 5-fold cross-validation has been employed. For 5-fold cross-validation, the dataset has been divided randomly into five equal subsets. One subset has been used for testing and the other subsets have been used for training machine learning models. Repeating this process five times, the average 3D accuracy map has been obtained to evaluate the local structural differences between ASD and TDC ultimately. Generally, 5-fold cross-validation also has been repeated several times to enhance the robustness of the results. The higher accuracy of the voxels have, the more significant structural changes around the voxels. Similar to the previous study (Feng et al., 2012), a rigorous threshold (70% in the present study) has been set to identify meaningful clusters (features) with a cluster size larger than 50 voxels. The brain regions involved in these clusters can be considered as candidate brain regions that are related to structural alteration.

### 3.2. Feature-Level Assessment

Feature-level assessment in the study has processed the ‘virtual lesion' analysis based on these candidate brain regions (Chang et al., 2015) involved in the above clusters. Originally, the “virtual lesion” analysis has been applied to investigate how individual regions or networks contribute to the prediction of ML models by removing or using each region or network at a time from the model based on a selected parcellation. Based on AAL parcellation, we have divided the clusters identified in model-level assessment into different brain regions and utilized the “virtual lesion” analysis to investigate their classification performance separately based on three different ML models including PCA+Ridge, PCA+SVM, and Bagging. For PCA+Ridge and PCA+SVM, PCA has been used to reduce the dimensionality of data, and Ridge/SVM has been used as the classifier, respectively.

#### 3.2.1. Ridge Classifier

Ridge method has been proposed to solve the regression problem originally by imposing a penalty on the coefficient vector ***w*** on the following objective function (Rifkin et al., 2003):


(10) $$\begin{array}{l} \underset{w}{\min}\|Xw-y\|{}_{2}^{2}+\alpha \|w\|{}_{2}^{2} \end{array}$$

where *X* is the dataset, *y* is the data label. The penalty factor α is used to control the amount of shrinkage. The larger the value of α, the greater the amount of shrinkage. When utilized for classification problems, the Ridge classifier converts binary targets *y* to {−1, +1} and treats them as regression tasks, optimizing the above objective function.

#### 3.2.2. Bagging Classifier

As a kind of ensemble algorithms, the bagging method has used a base estimator to build several instances from random subsets of the original training set and then average the predictions of these instances to drive a final prediction, which is helpful to reduce the variance of a base estimator. In this present study, the base estimator used a decision tree by default. Given training vectors $x_{i}\in {\mathbb{R}}^{n},\text{i}=1,\ldots m$ and the label vector *y* ∈ ℝ^*l*^, a decision tree employs a tree to model the classification problem, which partitions the feature space recursively to make samples with the same label grouped together. Supposed the data at node *m* be expressed by *Q*_*m*_ with *N*_*m*_ samples. For each candidate split θ = (*j, t*_*m*_) consisting of a feature *j* and threshold *t*_*m*_, partition training data into $Q_{m}^{left}\left(\theta \right)$ and $Q_{m}^{right}\left(\theta \right)$ subsets as follows:


(11) $$\begin{array}{l} Q_{m}^{left}\left(\theta \right)=\left\{\left(x,y\right)|x_{j}<=t_{m}\right\} \end{array}$$

(12) $$\begin{array}{l} Q_{m}^{\text{right}}\left(\theta \right)=Q_{m}\backslash Q_{m}^{\text{left}}\left(\theta \right) \end{array}$$

The “best” split has been determined according to the following objective function:


(13) $$\begin{array}{l} \theta^{*}={\mathrm{argmin}}_{\theta }G\left(Q_{m},\theta \right) \end{array}$$

where


(14) $$\begin{array}{l} G\left(Q_{m},\theta \right)=\frac{N_{m}^{left}}{N_{m}}H\left(Q_{m}^{left}\left(\theta \right)\right)+\frac{N_{m}^{\text{right}}}{N_{m}}H\left(Q_{m}^{\text{right}}\left(\theta \right)\right) \end{array}$$

and *H*(*Q*_*m*_) is the impurity function using Gini index to evaluate the performance of the candidate split whether they grouped samples with the same label into the same group:


(15) $$\begin{array}{l} H\left(Q_{m}\right)=\underset{k}{\sum }p_{mk}\left(1-p_{mk}\right) \end{array}$$

where *p*_*mk*_ is the probability of picking up a data point with class label *k* in node *m*. For subsets $Q_{m}^{\text{left}}\left(\theta^{*}\right)$ and $Q_{m}^{\text{right}}\left(\theta^{*}\right)$, the same procedure was executed recursively until the maximum depth is reached.

#### 3.2.3. Hyperparameter Tuning Based on Optuna

Since ML models are sensible to the setting of hyper-parameters, the hyper-parameter tuning technique based on Optuna has been employed (Akiba et al., 2019) to seek the optimal parameters for these models. With Optuna, the optimal percentage of the total variance in PCA has been searched from 0.6 to 0.99 with step 0.1. The optimal penalty parameter *C* of SVM and the optimal penalty coefficient α of Ridge have been searched from 10^−10^ to 10^10^ satisfied a uniform distribution in the log domain. For Bagging, the optimal number of the estimator has been searched from 3 to 30 with step 1. The optimal percentage of samples and features to draw from dataset *X* to train each base estimator has been searched from 0.5 to 1 with a uniform distribution in the linear domain.

### 3.3. Biology-Level Assessment

To explore the association between the regional GM volume reduction and the clinical severity of ASD, the study has performed correlation analysis of regional GM volume of candidate biomarkers with the clinical scores with ADI_R (the Autism Diagnostic Interview–Revised) (Lord et al., 1994) and ADOS_G (the Autism Diagnostic Observation Schedule) (Lord, 2000). ADI_R and ADOS_G are considered as the “gold standard” assessment measures in the evaluation of ASD. ADOS_G is a semi-structured, standardized assessment of communication, social interaction and play and imaginative use of materials for individuals. However, unlike ADOS_G, ADI_R is a comprehensive parent interview to measure social interaction, communication and language, and repetitive, restricted, and stereotyped interests and behavior. Scores assessed by ADOS_G and ADI_R are able to reflect the symptom severity of ASD. Meanwhile, the study also has compared these findings with previous literature in the section of “Discussion” to explore the neurobiological meaning of the structural alteration.

## 4. Experiments and Results

### 4.1. Experiments Setting

All experiments have been implemented on Ubuntu 16.4 with Python 3.7 and sklearn package 2.4. In the stage of feature-level assessment, 5-fold nested cross-validation has been conducted on GU dataset with ten iterations to evaluate the robustness and generalizability of ML models on individual candidate brain regions. For 5-fold nested cross-validation, it consists of an outer loop and an inner loop. During the outer loop, the dataset is split randomly into five equal subsets. Among these subsets, one subset is test data and the other subsets are training data. During the inner loop, the training data further is divided into five equal subsets, one subset is validation data and the rest subsets have been used to test the performance of different hyper-parameters. Therefore, the inner loop is used to tune the hyper-parameters, and the outer loop is used to estimate the model performance with optimal hyper-parameters. Besides, a multi-site validation also has been adopted, which trains ML models on GU and tests the predictive performance of the models using OHSU and UCLA. For each ML model of an individual brain region, the stability analysis has been conducted in terms of sensitivity, specificity, and permutation test. Sensitivity and specificity are two important metrics to measure the predictive ability of the ML model. Selecting the optimal balance between sensitivity and specificity depends on the purpose for which the test is used. The study has defined a threshold of 20% to quantify the differences between sensitivity and specificity, and a good balance between sensitivity and specificity should be less than the threshold. Furthermore, the permutation test (Ojala et al., 2010) was also used to evaluate the statistical significance of the predictive performance for each brain region. For the permutation test, the class labels of training data were randomly permuted and then 5-fold cross-validation was performed on the permuted training set. The permutation was repeated 5,000 times. During the permutation test, the statistical significance *p* is defined as the percentage of the accuracies that was equal to or greater than the accuracies obtained from the non-permuted data. Brain regions with *p* < 5% (*p* < 0.05) were considered statistically significant. Brain regions without a good balance between sensitivity and specificity and without statistical significance in permutation tests have been excluded from candidate brain regions. The final candidate brain regions have considered the structural biomarkers related to ASD.

### 4.2. The Results of Statistical Analysis

**Figure 2:**
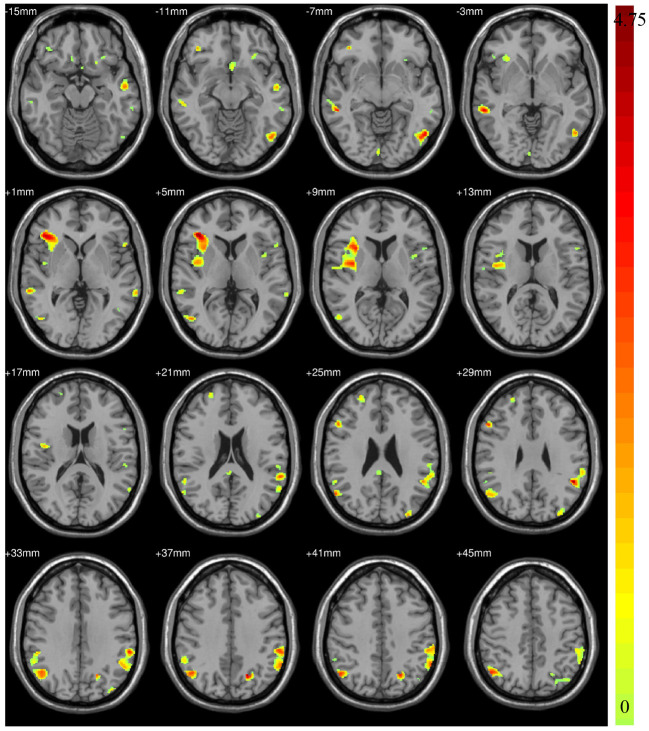
The decreased Gray Matter (GM) volume detected by statistical analysis.

The between-group differences found by the two-sample *t*-test on GU have been illustrated in Figure 2 and Table 3. It can be found that the atrophy of the GM volume is widespread covering the frontal lobe, parietal lobe, temporal lobe, occipital lobe, insula and the limbic system, especially nearby insula, temporal lobe, and inferior parietal lobule. The atrophy brain regions involved in brain function including visual information processing (e.g., BA18, BA19, and BA20), the language understanding, processing and representation and auditory processing (e.g., BA21, BA22, BA39, BA40, BA44, and BA47), emotion regulation (e.g., BA23), Olfactory function (e.g., BA25, BA28), and face recognition (e.g., BA37), cognitive function (e.g., BA10), visual-motor coordination (e.g., BA7), and emotional correlation (e.g., BA13). ASD patient's dysfunction to some extent probably means that the decrease of GM volume is related to ASD. In addition, there were no significant volumetric differences for WM.

**Table 3:** The different brain regions detected by statistical analysis on the GU dataset.

**Cluster**	**Brain regions**	**BA**	**Peak MNI coordinates(mm)**			**Cluster size**	**T**
**ID**			**x**	**y**	**z**	**(voxels)**	
1	MFG.R	-	51	43.5	10.5	83	3.949
2		10	27	52.5	25.5	223	3.8232
3		-	33	39	25.5	99	3.4774
4		-	28.5	30	48	80	3.6508
5	ORBinf.L	-	–42	34.5	-19.5	83	3.4724
6		-	–18	36	–15	68	3.6211
7		-	–40.5	51	–4.5	58	3.4671
8	IFGoperc.L	47	-19.5	18	-21	707	3.7632
9	CAL.L,LING.L	18	4.5	–88.5	–6	221	3.7435
10	SFGdor.R	-	28.5	3	58.5	55	3.5629
11	IFGtriang.R, IFGoperc.R	-	52.5	19.5	27	159	4.3846
12	MTG.L	-	–67.5	–40.5	3	100	4.1571
13		-	–54	–9	–13.5	448	4.2016
14	MTG.R	37	48	–70.5	4.5	259	4.1095
15		21,22	54	-39	–6	912	4.7229
16	INS.L,IFGoperc.L, IFGtriang.L	44	–55.5	18	1.5	395	3.8977
17	ITG.L	20,37,21	–51	–39	–22.5	594	3.9155
18	FFG.R	-	30	–12	–39	138	3.708
19		20	42	–37.5	–25.5	189	3.9689
20	SMG.L,IPL.L, ANG.L,MOG.L, STG.L,SOG.L, SPG.L	40,39,19, 7,22	–19.5	–64.5	36	4,006	4.6061
21	ANG.R,SMG.R, IPL.R,MOG.R	40,39,19, 7,22	51	–64.5	42	2,081	4.3024
22	IOG.L,ITG.L	19,37	-52.5	–66	–6	809	4.4788
23	PCG.R	23	3	–39	22.5	129	3.6955
24	OLF.L,REC.R, OLF.R	25	–1.5	13.5	–12	674	3.7786
25	PHG.R	28	18	–19.5	–21	289	4.0787
26	INS.R,ROL.R,ORBinf.R, IFGtriang.R,IFGoperc.R	13,44,47	42	30	3	2998	4.7535

### 4.3. The Results of Model-Level Assessment

**Figure 3:**
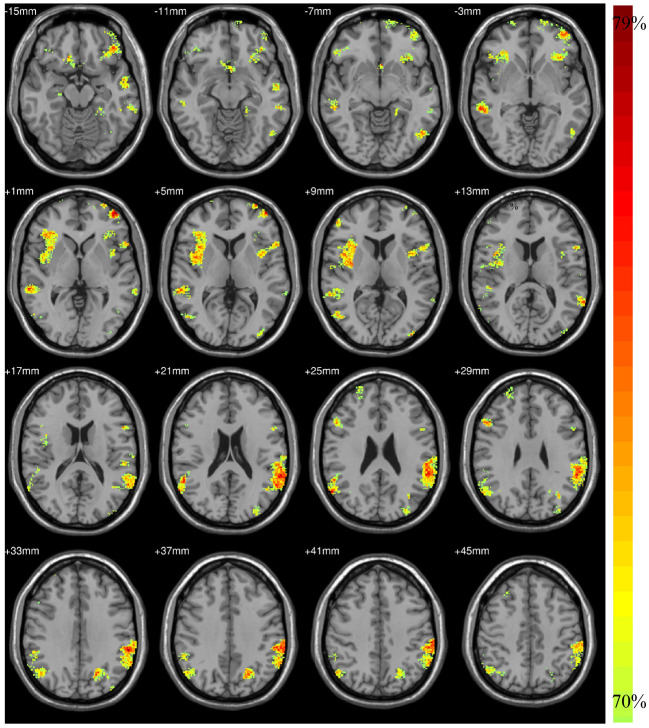
The structural alteration by model-level analysis.

According to the findings in model-level assessment based on the searchlight method, the study has found structural differences of GM within twenty-four clusters, as shown in Figure 3 and Table 4. It can be seen that these clusters have covered most brain regions found by traditional statistical analysis, except FFG.R,CAL.L,INS.L, bilateral MOG, and PCG.R. For these brain regions failed to be detected in model-level assessment, the possible reason is that the differences are not obvious, and the areas of the clusters containing these brain regions are small in statistical analysis, e.g., the cluster of CAL.L only has 93 volxes, MOG.R and PCG.R only have 63 and 56 voxels, respectively. Significantly, although the peak MNI coordinates of some clusters may be different, model-level assessment and statistical analysis have detected similar brain regions, such as cluster 20 (in Table 3) and cluster 19 (in Table 4), cluster 21 (in Table 3) and cluster 14 (in Table 4), cluster 26 (in Table 3) and cluster 13 (in Table 4), cluster 10 (in Table 3) and cluster 24 (in Table 4), cluster 11 (in Table 3) and cluster 20 (in Table 4). Furthermore, we have considered abnormal clusters identified by the model-level assessment as the features and classified them on GU and multi-site data. The good classification performances have been shown in Supplementary Tables 6, 7 separately to demonstrate the effectiveness of these abnormal clusters.

**Table 4:** The candidate brain regions detected by model-level assessment.

**Cluster**	**Brain regions**	**BA**	**Peak MNI coordinates (mm)**			**Cluster size**	**Classification**
**ID**			**x**	**y**	**z**	**(voxels)**	**Accuracy (%)**
1	ITG.L,MTG.L	37	–49.5	–36	–19.5	396	74.6
2	ITG.L,MTG.L,IOG.L	-	–54	–66	–7.5	243	75.4
3	MTG.L	-	–57	–10.5	–15	285	75.9
4		-	–69	–40.5	3	59	74.5
5	MTG.R	39	48	–70.5	7.5	194	75.1
6	PHG.L, HIP.L	28	–19.5	–18	–21	183	74.3
7	ORBinf.L	-	–18	18	–25.5	217	75.4
8	ORBsup.R,REC.R, OLF.L,OLF.R	47	16.5	25.5	–19.5	819	76.9
9	PHG.R,HIP.R	-	18	–21	–21	305	75.4
10	Crus1.L	-	–36	–70.5	–21	99	74.2
11	FFG.L	20	–33	–36	–21	191	74.6
12	ORBinf.L,ORBmid.L, MFG.L,INS.L, IFGtriang.L,ORBsup.L	10,11,47	–43.5	54	0	2068	78.1
13	INS.R,ROL.R, IFGoperc.R,ORBinf.R, IFGtriang.R	13,47,44	31.5	30	1.5	2,499	76.6
14	ANG.R,MTG.R, STG.R,SMG.R, IPL.R	40,39, 22,7	60	-36	0	2,634	78.6
15	IFGoperc.L,INS.L, IFGtriang.L	44,13	–34.5	6	6	682	75.6
16	MOG.L	19	-37.5	–91.5	9	119	74.6
17		19	–30	-85.5	25.5	192	73.8
18	IFGtriang.R,MFG.R	-	51	43.5	10.5	107	75.4
19	SMG.L, IPL.L, STG.L, MTG.L, ANG.L,PoCG.L	40,22, 39,2	–58.5	–37.5	33	4,244	78.3
20	IFGoperc.R, IFGtriang.R	-	52.5	19.5	27	176	76.2
21	MFG.R	10	22.5	63	27	128	72.8
22		-	28.5	33	48	59	73.6
23	MOG.L,SOG.L, SPG.L	7	–18	–69	36	578	76.4
24	SFGdor.R	-	28.5	1.5	57	102	73.5

### 4.4. The Results of Feature-Level Assessment

**Figure 4:**
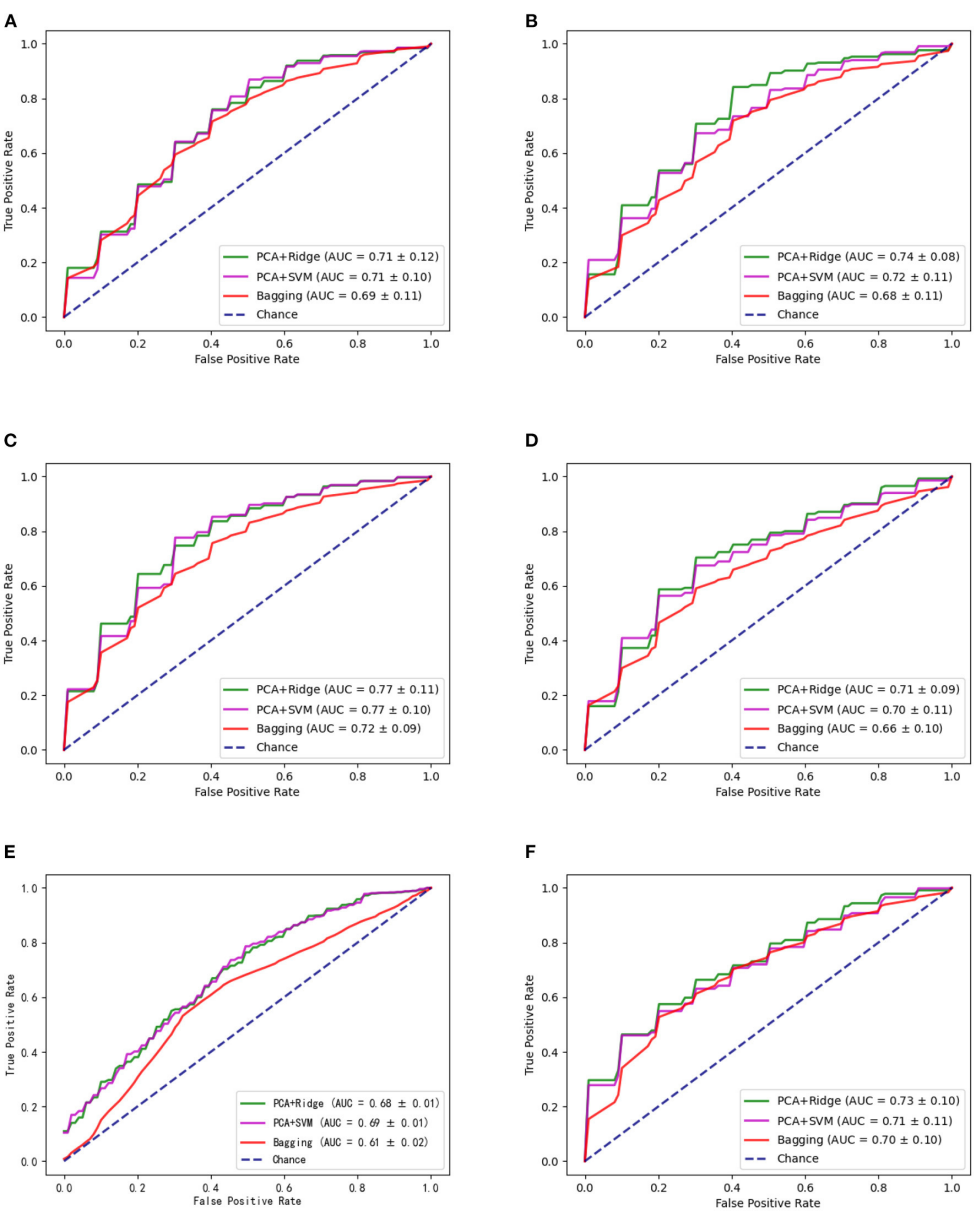
The ROC curves of different methods for candidate biomarkers including ORBinf.L (left opercular part of inferior frontal gyrus) **(A)**, STG.L (left superior temporal gyrus) **(B)**, SMG.L (left supramarginal gyrus) **(C)**, SMG.R (right supramarginal gyrus) **(D)**, ANG.L (left angular gyrus) **(E)**, and ANG.R (right angular gyrus) **(F)**.

For the candidate brain regions detected in model-level assessment, the 'virtual lesion' analysis has been further conducted to select robust and discriminant brain regions which can be considered the neuroimaging biomarkers of ASD. The classification performances of final selected brain regions with nested cross-validation and multi-site validation have been listed in Tables 5, 6 separately. The bold values are the best performance for these brain regions. We have also illustrated the ROC curves of candidate biomarkers for different ML models on the GU dataset with the best accuracies larger than 70% in Figure 4, which include ORBinf.L,STG.L,SMG.L,SMG.R,ANG.L,ANG.R. The ROC curves of other candidate biomarkers on GU and multi-site data have been provided in the Supplementary Material.

**Table 5:** The classification performance on GU.

**Brain region**	**PCA+Ridge**				**PCA+SVM**				**Bagging**			
	**ACC**	**SEN**	**SPE**	***p***	**ACC**	**SEN**	**SPE**	***p***	**ACC**	**SEN**	**SPE**	***p***
	**(%)**	**(%)**	**(%)**		**(%)**	**(%)**	**(%)**		**(%)**	**(%)**	**(%)**	
IFGoperc.L	**65.02**	**66.73**	**63.64**	0.003	65.06	60.73	69.09	0.018	62.29	65.09	60.00	0.016
IFGoperc.R	66.02	60.55	70.91	0.007	67.92	60.73	74.55	0.008	**66.15**	**64.55**	**67.27**	0.007
ORBinf.L	67.01	62.91	70.91	0.0013	**71.77**	**62.91**	**80.00**	0.0003	68.92	68.73	69.09	0.0001
ROL.R	64.11	62.73	65.45	0.003	**65.11**	**66.91**	**63.64**	0.0005	62.34	63.09	61.82	0.006
OLF.R	66.10	62.91	69.09	0.003	68.18	63.63	72.72	0.0009	**69.96**	**68.73**	**70.91**	0.008
REC.R	64.24	61.27	67.27	0.001	**66.66**	**70.00**	**63.63**	0.008	63.38	66.73	60.00	0.001
INS.R	**68.83**	**62.55**	**74.55**	0.0005	67.97	61.09	74.55	0.0009	67.92	66.55	69.09	0.004
IPL.L	67.92	68.55	67.27	0.0003	**67.97**	**66.91**	**69.09**	0.006	63.2	66.55	60.00	0.001
SMG.L	**76.32**	**72.55**	**80.00**	0.0001	75.41	70.73	80.00	0.0001	69.74	70.55	69.09	0.0005
SMG.R	**70.74**	**70.55**	**70.91**	0.0001	71.65	64.73	78.18	0.001	65.15	62.91	67.27	0.005
ANG.L	65.08	63.33	66.67	0.008	**72.72**	**81.81**	**63.63**	0.003	63.25	66.73	60.00	0.004
ANG.R	69.91	66.73	72.73	0.001	69.00	62.91	74.55	0.009	**71.73**	**76.36**	**67.27**	0.001
STG.L	**70.78**	**66.73**	**74.55**	0.0001	70.78	61.09	80.00	0.001	67.88	64.73	70.91	0.007
STG.R	65.15	60.73	69.09	0.001	67.06	62.91	70.91	0.0009	**66.02**	**66.73**	**65.45**	0.007
MTG.L	72.72	81.81	63.63	0.008	61.90	60.00	63.63	0.006	**68.01**	**66.91**	**69.09**	0.0001
MTG.R	62.21	62.73	61.82	0.003	66.06	60.91	70.91	0.002	**68.83**	**68.55**	**69.09**	0.002
ITG.L	62.39	60.68	63.64	0.01	**66.15**	**60.73**	**70.91**	0.01	64.2	66.73	61.82	0.001

*ACC, Accuracy; SEN, Sensitivity; SPE, Specificity; p-The significant level of permutation test*.

**Table 6:** The classification performance on the multi-site dataset (OHSU and UCLA).

**Brain region**	**PCA+Ridge**				**PCA+SVM**				**Bagging**			
	**ACC**	**SEN**	**SPE**	***p***	**ACC**	**SEN**	**SPE**	***p***	**ACC**	**SEN**	**SPE**	***p***
	**(%)**	**(%)**	**(%)**		**(%)**	**(%)**	**(%)**	**(%)**	**(%)**	**(%)**		
IFGoperc.L	62.40	62.26	62.50	0.004	**63.20**	**60.38**	**65.28**	0.005	61.60	60.38	62.50	0.0009
IFGoperc.R	64.80	62.26	66.67	0.0009	65.60	54.72	73.61	0.004	**65.60**	**62.26**	**68.06**	0.001
ORBinf.L	64.00	66.04	62.50	0.0002	61.60	60.38	62.50	0.002	**68.00**	**71.70**	**65.28**	0.001
ROL.R	65.60	56.60	72.22	0.0002	**70.4**	**60.38**	**77.78**	0.0001	68.80	62.26	73.61	0.002
OLF.R	**66.40**	**60.38**	**70.83**	0.002	60.80	62.26	59.72	0.0007	60.87	60.47	61.11	0.009
REC.R	**67.20**	**60.38**	**72.22**	0.0009	65.60	60.38	69.44	0.007	63.20	60.38	65.28	0.006
INS.R	66.40	56.60	73.61	0.0003	68.70	60.38	73.61	0.0001	**68.80**	**66.04**	**69.44**	0.0001
IPL.L	**70.40**	**66.04**	**73.61**	0.0002	69.60	64.15	73.61	0.007	68.80	64.15	72.22	0.0007
SMG.L	64.80	58.49	69.44	0.0001	**69.57**	**62.79**	**73.61**	0.0001	66.40	62.26	69.44	0.008
SMG.R	**66.40**	**73.58**	**61.11**	0.0001	66.40	71.70	62.50	0.0003	65.60	67.92	63.89	0.01
ANG.L	64.35	65.12	63.89	0.0002	**66.4**	**60.38**	**70.83**	0.0009	66.40	62.26	69.44	0.01
ANG.R	60.80	60.38	61.11	0.001	**66.09**	**62.79**	**68.06**	0.008	60.87	62.79	59.72	0.0009
STG.L	**68.80**	**66.04**	**70.83**	0.0002	67.20	62.26	70.83	0.007	66.40	67.92	65.28	0.0001
STG.R	**72.80**	**69.81**	**75.00**	0.0002	72.00	67.92	75.00	0.0002	67.20	69.81	65.27	0.0003
MTG.L	64.35	62.80	65.28	0.0007	65.22	60.47	68.06	0.005	**64.80**	**64.15**	**65.28**	0.0007
MTG.R	68.00	66.04	69.44	0.0001	**68.80**	**66.04**	**70.83**	0.0002	67.20	66.04	68.06	0.0001
ITG.L	65.60	67.92	63.89	0.0002	66.96	60.47	70.83	0.0005	**66.96**	**65.12**	**68.06**	0.04

*ACC, Accuracy; SEN, Sensitivity; SPE, Specificity; p-The significant level of permutation test*.

### 4.5. Neuroanatomical Correlations Between Regional GM Volume and Symptom Severity

We have processed correlation analysis to assess the relationship between the regional GM volume of candidate biomarkers in the GU dataset and ASD symptom severity. Clinical scores(ADOS_G and ADI_R) of thirty-six participants are available in GU. Results have revealed negative correlations between the communication skill scores in ADOS_G and regional GM volume in gyrus rectus (*r* = −0.356, *p* < 0.05), left middle temporal gyrus (*r* = −0.330, *p* < 0.05), and left inferior temporal gyrus (*r* = −0.339, *p* < 0.05). In particular, a significant negative correlation has been found between the communication skill scores in ADOS_G and the orbital part of the left inferior frontal gyrus (*r* = −0.433, *p* < 0.01). For ADI_R scores, we also found a negative correlation between verbal skill scores and right angular gyrus (*r* = −0.344, *p* < 0.05) and a significant negative correlation between non-verbal communication skills and right angular gyrus (*r* = −0.424, *p* < 0.01). No significant positive correlation between regional GM volume and clinical scores was found.

## 5. Discussion

Current VBM findings have delineated brain regions with consistently increased or reduced GM volume (Cauda et al., 2011). In this study, statistical analysis has reported a widespread reduction of GM volume in ASD with 7-13 years old. Research on brain development in ASD across the lifespan has demonstrated a complex neurodevelopmental trajectory, characterized by an early brain overgrowth followed by undergrowth in middle childhood and early adolescence (Courchesne et al., 2001). This might support the findings of statistical analysis in our study.

Our results based on machine learning have demonstrated that a widespread structural alteration of GM volume involved in bilateral superior temporal gyrus, bilateral middle temporal gyrus, left inferior temporal gyrus, right orbital SFG, bilateral opercular inferior frontal gyrus, left orbital inferior frontal gyrus, right rolandic operculum, right olfactory cortex, right gyrus rectus, right insula, right inferior parietal lobe with Supramarginal gyrus and Angular gyrus. Especially, multi-site dataset validation also has verified the robustness of the machine learning framework with three-level assessment. Since ASD is a complex neurodevelopmental disorder, involving language, reading, emotion, social interaction impairments, the quantitative meta-analysis in Geschwind and Levitt (2007) and Maximo et al. (2014) have suggested that ASD is unlikely to be associated with the abnormalities in one specific region alone but to be linked to the abnormalities of multiple, spatially distributed, neural systems. The finding may shed light on the widespread differences in GM volume found in our study.

The findings in the study have almost covered the whole temporal lobe including bilateral superior temporal gyrus, bilateral middle temporal gyrus, and left inferior temporal gyrus. Since attention has been directed to explore the neurobiological mechanism of ASD first, the abnormality of the temporal lobe has been speculated to link with the deficits in language and social behavior of patients with ASD (Hauser et al., 1975; Bachevalier, 1994; Kates et al., 2010). Ritvo et al. (1986) has examined the brains of four autistic subjects and found the localized pathological changes in the temporal lobe from autopsy-based research. It is considered that the superior temporal gyrus is a potential import biomarker of ASD (Pierce, 2011; Sophia et al., 2013). Based on the VBM-Dartel technique, Riddle et al. (2016) have revealed enlargement of the left anterior superior temporal gyrus in ASD. It is believed (Bigler et al., 2007) that the superior temporal gyrus plays a crucial role in social cognition, which participates in auditory and language processing. On the other hand, the VBM analysis has found reduced GM volume in the middle temporal gyrus (Kohoutov et al., 2020). According to analyze structural images of low functioning ASD children from 2 to 10 years old, the reduction of GM volume in the left inferior temporal gyrus has been identified, which appears to be involved in visual object perception (Riva et al., 2013). Similarly, RT et al. (2000) have revealed the fMRI (functional MRI) alterations of the inferior temporal gyrus when engaging in facial recognition tasks. Zilbovicius et al. (2000) has also found the localized dysfunction of the temporal lobe from the aspect of PET. Brothers (2002) has proposed the concept of the social brain first in 1990, which was defined as a group of interrelated neuroanatomical structures which are used to process social information, recognize other individuals and evaluate their psychological state, including intentions, dispositions, desires, and beliefs. The temporal lobe plays a very important role in the hypothesis of the social brain. The posterior superior temporal sulcus recognizes biological movements, such as eyes, hands, and other body movements and helps to interpret and predict others' behavior and intentions (Allison et al., 2000). The fMRI study of patients with ASD has shown that the differences in the activation on temporal lobe compared with their families and normal people, and the worse the social ability, the weaker the activation. Furthermore, it has been found that the degree of activation was positively correlated with the clinical manifestations of social impairment (Sugrue et al., 2010). The abnormality of the temporal lobe found in this study not only supports the hypothesis of the social brain but also suggests that the area of temporal lobe abnormalities in patients with autism may be larger than that found in previous studies. However, the hypothesis still needs to be further confirmed by quantitative and qualitative autopsy reports and animal studies.

Furthermore, the study has found GM abnormalities in the Broca area (posterior frontal lobe corresponding to BA44) and Wernicke area (superior marginal gyrus corresponding to BA39 and angular gyrus corresponding to BA40). Meanwhile, we also found negative correlations between GM volume in the right angular gyrus and verbal/nonverbal communication score in ADI_R. Language deficits are the core diagnostic characteristics in ASD and both of Broca area and Wernicke area are associated with language understanding. Adam et al. (2004) found that the Wernicke area, which is responsible for the understanding of single words, is more active than the Broca area, which is responsible for the understanding of complex sentences and has proposed the “underconnectivity theory” to explain why some patients with ASD have excellent ability to process single words, rather than complex sentences. Osbarn (2020) has found weakened functional connectivity in the area of Wernicke. Recently, researchers have established “the broken mirror theory” of autistic patients. It is supported that the dysfunction of the Human Mirror Neuron System (MNS) is the main cause of social and cognitive deficits in ASD (Vivanti and Rogers, 2014). The Broca area in humans has been considered as homologous to F5 as a part of MNS. The results in our studies also supported the MNS dysfunction in ASD individuals. However, the relevant evidence about the role of MNS in ASD still is not enough which urges us to build a more perfect MNS theory to understand the causes of social communication disorder in ASD (Southgate and Hamilton, 2008).

For other regions identified in the present study, they have also been reported in previous literature. Shijun (2021) has constructed a three-dimensional residual network based on deep learning and found the GM reduction in the orbital inferior frontal gyrus and Rolandic operculum. Riva et al. (2013) also found the reduced GM volume in the orbital part of the inferior frontal gyrus. In light of the meta-analysis based on large samples, the volume of GM in the insula and inferior parietal lobe decreased (Cauda et al., 2011). Li et al. (2019) have found that the GM volume of the bilateral gyrus rectus decreased, and the left rectus was negatively correlated with the clinical symptom score. Although it is not reported that the volume changes in the olfactory cortex of patients with ASD, the olfactory cortex is located at the anterior bottom of the limbic system and reciprocally connected with other structures, such as the amygdala, hippocampus, hypothalamus, the olfactory cortex is related to emotion and memory. As a consequence, the abnormal GM in the olfactory cortex in ASD may lead to emotional and memory problems. It is worth mentioning that some current studies on ASD have found GM differences in the cerebellum, hippocampus, and parahippocampal gyrus (Faridi and Khosrowabadi, 2017; Lotze et al., 2019). In particular, a decreased number of Purkinje cells in the cerebellar hemisphere have been observed (Ritvo et al., 1986). The study only found that the differences of the parahippocampal gyrus during statistical analysis. For model-level assessment, we have detected the alterations in these three brain regions, including the bilateral hippocampus, and parahippocampal gyrus and left crus in the cerebellum. However, they have been excluded from the candidate brain regions due to the poor performance in stability analysis. Traut et al. (2018) have compared and analyzed cerebellar volume of a large sample of ASD patients with normal subjects, and reported that the change of the cerebellar volume was significantly correlated with age, gender, and IQ rather than ASD diagnosis. Even though some studies have reported the differences of WM in ASD (Ecker et al., 2010; Xiao et al., 2014; Górriz et al., 2019), our study has not found abnormal white matter based on structural images. At present, contradictory conclusions often have been derived from a variety of ASD research due to the heterogeneity of subjects, including different subtypes, different scanning parameters from different centers, different ages and genders in ASD (Pua et al., 2017; Hiremath et al., 2021). In addition, the inconsistent findings of research work based on machine learning might be induced by different feature exaction techniques. For example, Haar et al. (2016) have achieved poor classification based on morphological features of ROI on the multi-site dataset and suggested that anatomical abnormalities may be only present in some distinct subgroups of ASD, while Zheng et al. (2018) have obtained superior classification performance with multi-feature-based networks based on morphological features.

Our results suggest that structural MRI can provide neuroimaging-based biomarkers for ASD. Such biomarkers could be used to complete and improve the diagnosis and treatment of ASD clinically (Walsh et al., 2011). On the one hand, we can utilize the classification performance of these identified biomarkers based on the machine learning model proposed in the study to improve diagnostic accuracy. On the other hand, the behavioral social malfunctioning in ASD might be modified by neural or behavioral treatments. For example, it is also reported that the behavioral training of facial expression communication behavior can help to improve the neural activities of ASD patients related to some social brain regions, such as MTG, so as to improve their capability of expression recognition (Bölte et al., 2015).

Although our results are consistent with some previous reports, several limitations of our study should be acknowledged. First, the confounding factors should be considered. ASD is a complex disease with multiple confounding factors, such as age, gender, IQ, and the inherent heterogeneity of the disorder. Our studies attempted to control age and gender in statistical analysis but failed to find an appropriate approach to remove the influence of the confounding factors in machine learning methods. On the other hand, the controlling of confounding factors is still highly controversial in the studies of ASD (Thomaidis et al., 2015). Some researchers have claimed that IQ should be strictly matched or statistically regressed out, while others have argued that the variability truly associated with ASD could be also discarded as “non-specific” when attempting to control some non-specific factors, such as IQ (Osbarn, 2020). Some studies have investigated the gender differences in ASD (Halladay et al., 2015; Prosperi et al., 2021) due to a high incidence rate of ASD in boys. Second, the current study took advantage of the relatively large sample of participants with ASD in the ABIDE II database to process multi-site validation *via* machine learning. However, we have to point that all three databases used in our study were collected by Siemens scanner with similar scanning parameters, which might make a less dispersion among data. In the future, the investigation of ASD about neuroanatomical alterations on larger samples from diverse clinical and demographic subgroups will significantly promote understanding neuropathology mechanism in ASD.

## 6. Conclusion

In this study, the VBM analysis has revealed a widespread reduction of GM volume when comparing ASD with TDC. Furthermore, our machine learning analysis followed the unified machine learning framework has revealed candidate neuroimaging biomarkers related to ASD and confirmed the relationship between regional GM volume and symptom severity. Our results have suggested that candidate neuroimaging biomarkers are useful to characterize the profile of brain anatomy in ASD and improve the diagnosis performance in clinical applications.

## Data Availability Statement

Publicly available datasets were analyzed in this study. This data can be found here: http://fcon_1000.projects.nitrc.org/indi/abide/abide_II.html.

## Author Contributions

YD, WZ, and CLi conceived and designed this study. HJ, YT, and XL participated in the analysis of MRI dataset. CLu and DY helped to improve the manuscript. All authors contributed to the article, read, and approved the final manuscript.

## Funding

This work is supported by the China Postdoctoral Science Foundation (No. 2016M592656), Sichuan Science and Technology Program (No. 2018JY0272), Science & Technology Bureau of Chengdu (No. 2020-YF09-00005-SN), Erasmus+ SHYFTE Project (No. 598649-EPP-1-2018-1-FR-EPPKA2-CBHE-JP), and the Key Laboratory of Pattern Recognition and Intelligent Information Processing, Institutions of Higher Education of Sichuan Province (No. MSSB-2021-12).

## Conflict of Interest

The authors declare that the research was conducted in the absence of any commercial or financial relationships that could be construed as a potential conflict of interest.

## Publisher's Note

All claims expressed in this article are solely those of the authors and do not necessarily represent those of their affiliated organizations, or those of the publisher, the editors and the reviewers. Any product that may be evaluated in this article, or claim that may be made by its manufacturer, is not guaranteed or endorsed by the publisher.
