# Supplementary material for: Identifying and Predicting Autism Spectrum Disorder Based on Multi-Site Structural MRI With Machine Learning
Source: Front Hum Neurosci. 2022 Feb 22;15:765517. doi: 10.3389/fnhum.2021.765517 (PMC8902595; doi:10.3389/fnhum.2021.765517)
Supplement: Supplementary file 1 [file Data_Sheet_1.PDF]

Supplementary Material

1 SUPPLEMENTARY TABLES AND FIGURES

1.1 Table

Table S1. Regions of interest (ROIs) included in the AAL-atlas.

Regions	Abbreviations	Regions	Abbreviations
Superior frontal gyrus, dorsolateral	SFGdor	Superior frontal gyrus, orbital part	ORBsup
Middle frontal gyrus	MFG	Middle frontal gyrus, orbital part	ORBmid
Inferior frontal gyrus, opercular part	IFGoperc	Inferior frontal gyrus, triangular part	IFGtriang
Inferior frontal gyrus, orbital part	ORBinf	Rolandic operculum	ROL
Supplementary motor area	SMA	Olfactory cortex	OLF
Superior frontal gyrus, medial	SFGmed	Superior frontal gyrus, medial orbital	ORBsupmed
Gyrus rectus	REC	Insula	INS
Anterior cingulate and paracingulate gyri	ACG	Median cingulate and paracingulate gyri	DCG
Posterior cingulate gyrus	PCG	Hippocampus	HIP
Parahippocampal gyrus	PHG	Amygdala	AMYG
Calcarine fissure and surrounding cortex	CAL	Cuneus	CUN
Lingual gyrus	LING	Superior occipital gyrus	SOG
Middle occipital gyrus	MOG	Inferior occipital gyrus	IOG
Fusiform gyrus	FFG	Postcentral gyrus	PoCG
Superior parietal gyrus	SPG	Inferior parietal, but supramarginal and angular gyri	IPL
Supramarginal gyrus	SMG	Angular gyrus	ANG
Precuneus	PCUN	Paracentral lobule	PCL
Caudate nucleus	CAU	Lenticular nucleus, putamen	PUT
Lenticular nucleus, pallidum	PAL	Thalamus	THA
Heschl gyrus	HES	Superior temporal gyrus	STG
Temporal pole: superior temporal gyrus	TPOsup	Middle temporal gyrus	MTG
Temporal pole: middle temporal gyrus	TPOmid	Inferior temporal gyrus	ITG
substantia nigra	SN	Red nuclei	RN

1.2 Figures

Table S2. The different brain regions detected by statistical analysis on OHSU dataset

Cluster ID	Brain Regions	BA	Peak MNI Coordinates(mm) x	y	z	Cluster Size (voxels)	T
1	ANG.R,IPL.R	40	48	-58.5	45	972	5.8416
2	PCG.R	23	6	-21	34.5	113	3.6869

Table S3. The different brain regions detected by statistical analysis on UCLA dataset

Cluster ID	Brain Regions	BA	Peak MNI Coordinates(mm) x	y	z	Cluster Size (voxels)	T
1	MTG.L,MOG.L	39	-46.5	-70.5	12	690	4.632
2	PreCG.L	6,9	-54	6	33	603	4.9082
3	SMA.R	6,9	7.5	-15	60	96	4.1358

Table S4. The classification performance on dataset from single site(GU) based on proposed framework

Brain Region	PCA+Ridge			PCA+SVM			Bagging		
	F1 (%)	Precision (%)	Recall (%)	F1 (%)	Precision (%)	Recall (%)	F1 (%)	Precision (%)	Recall (%)
IFGoperc.L	64.27	65.95	64.00	65.95	63.07	70.55	58.34	58.74	59.64
IFGoperc.R	63.86	64.39	65.55	65.69	63.81	70.00	63.57	64.12	65.82
ORBinf.L	66.54	66.15	67.82	72.02	67.41	78.18	61.81	64.02	61.09
ROL.R	61.19	64.58	59.82	62.78	64.11	63.64	61.93	60.95	64.36
OLF.R	65.37	65.03	66.00	66.22	61.04	72.55	63.57	63.16	65.27
REC.R	62.41	61.75	64.55	67.88	61.61	76.91	58.14	60.30	57.45
INS.R	68.1	68.02	69.64	69.31	64.08	76.73	64.55	62.54	68.18
IPL.L	64.32	64.52	66.36	65.18	64.81	67.45	60.1	61.58	60.55
SMG.L	75.47	74.05	78.36	75.08	72.80	78.91	65.82	67.55	66.00
SMG.R	69.83	74.07	70.00	69.34	72.48	70.91	59.86	62.52	60.00
ANG.L	60.28	60.70	60.36	64.47	59.67	70.91	59.00	59.19	59.82
ANG.R	65.14	65.37	65.64	66.01	64.96	68.00	64.43	67.48	63.64
STG.L	66.35	65.64	67.64	68.69	65.71	73.09	64.25	64.79	64.73
STG.R	62.67	62.23	64.18	64.64	61.25	69.64	60.01	60.57	60.73
MTG.L	64.86	61.83	69.09	64.72	60.52	70.73	63.37	63.69	64.00
MTG.R	63.69	62.64	65.45	64.93	60.15	71.64	65.91	65.48	66.91
ITG.L	60.69	60.36	62.00	64.23	58.85	71.64	60.47	62.21	60.18

Table S5. The classification performance on multi-site dataset(OHSU and UCLA) based on proposed framework

Brain Region	PCA+Ridge			PCA+SVM			Bagging		
	F1 (%)	Precision (%)	Recall (%)	F1 (%)	Precision (%)	Recall (%)	F1 (%)	Precision (%)	Recall (%)
IFGoperc.L	63.87	70.77	58.19	68.82	69.42	68.89	66.73	67.91	65.69
IFGoperc.R	69.16	67.92	70.56	71.93	67	78.06	65.8	69.74	62.78
ORBinf.L	65.91	71.01	61.53	70.28	69.38	71.94	64.3	69.55	60.14
ROL.R	71.21	71.32	71.11	72.43	71.74	73.19	70.63	71.79	69.58
OLF.R	69.49	71.8	67.36	70.07	70.27	70	60.08	68.67	53.75
REC.R	66.68	68.47	65.14	70.52	69.15	72.22	62.78	67.25	59.03
INS.R	73.59	70.78	76.67	74.93	70.42	80.14	62.98	68.15	58.89
IPL.L	71.4	73.7	69.31	73.64	73.12	74.31	67.21	71.5	63.75
SMG.L	69.24	70.35	68.19	69.64	71.16	68.19	67.63	71.84	64.03
SMG.R	66.65	76.92	58.89	65.4	73.76	58.89	62.49	70.54	56.25
ANG.L	65.28	71.84	60	70.47	70.02	72.08	65.67	69.34	62.5
ANG.R	64.52	69.69	60.14	67.9	70.86	65.42	65.25	70.31	61.39
STG.L	70.83	72.03	69.86	69.53	72.62	67.08	67.88	75.41	62.22
STG.R	71.61	78.75	65.69	74.65	77.99	71.81	72.44	77.73	67.92
MTG.L	69.27	73.99	65.14	71.24	72.36	70.56	64.91	72.38	59.03
MTG.R	72.19	72.16	72.36	68.36	69.38	67.78	65.53	67.45	64.03
ITG.L	68.17	67.41	69.03	71.07	69.9	72.64	66.78	73.78	61.25

Table S6. The classification performance on GU based on abnormal clusters identified by model-level assessment

	ACC(%)	SEN(%)	SPE(%)
PCA+Rdige	72.61	66.09	78.72
PCA+SVM	72.71	64.32	80.54
Bagging	66.05	64.04	67.81

Table S7. The classification performance on OHSU and UCLA based on abnormal clusters identified by model-level assessment

	ACC(%)	SEN(%)	SPE(%)
PCA+Rdige	63.44	50.94	72.64
PCA+SVM	63.60	52.64	71.66
Bagging	64.24	60.19	67.22

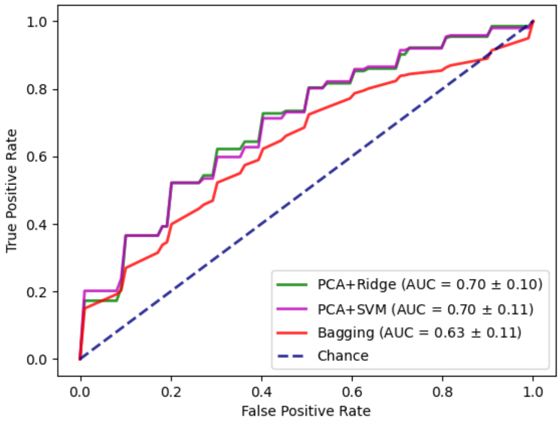

(1a) IFGoperc.L

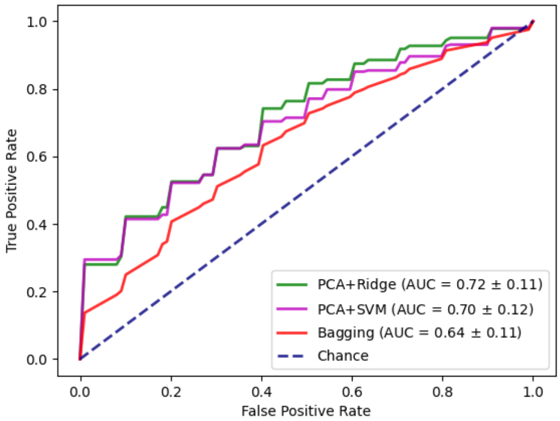

(1b) IFGoperc.R

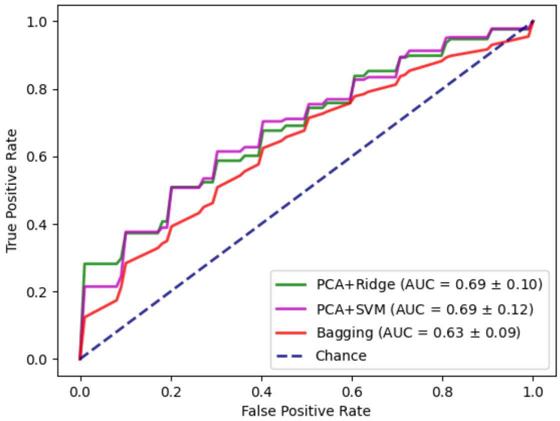

(1c) ROL.R

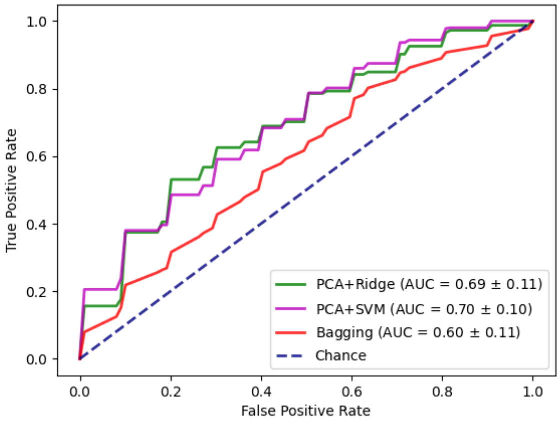

(1d) OLF.R

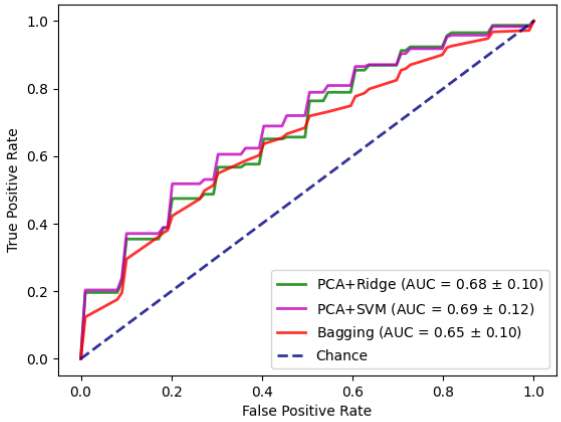

(1e) REC.R

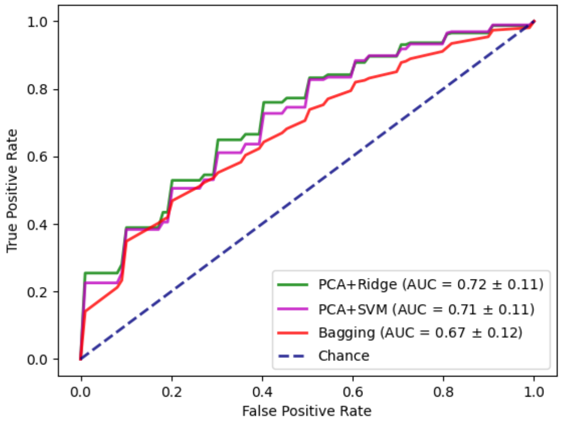

(1f) INS.R

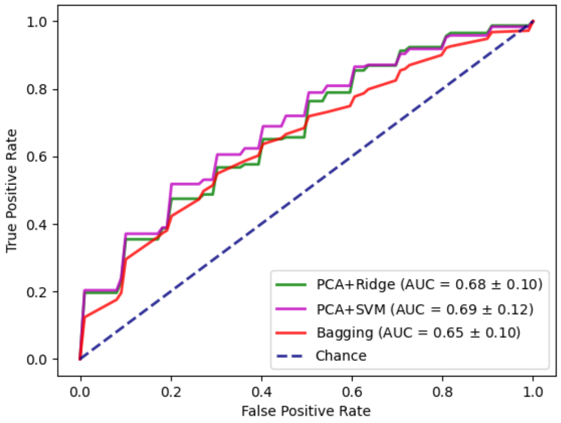

(1g) REC.R

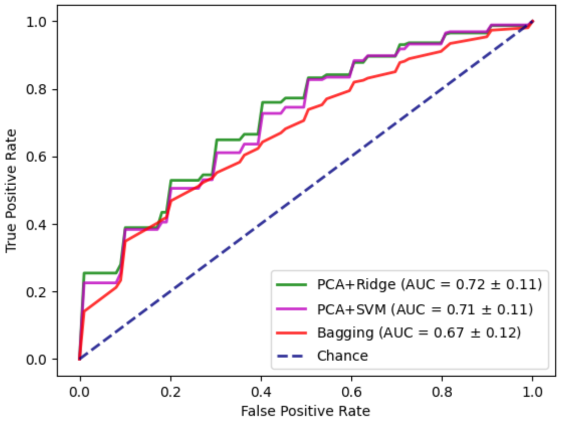

(1h) INS.R

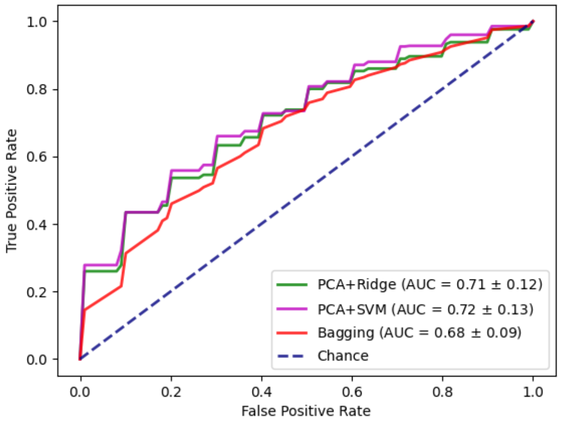

(1i) IPL.L

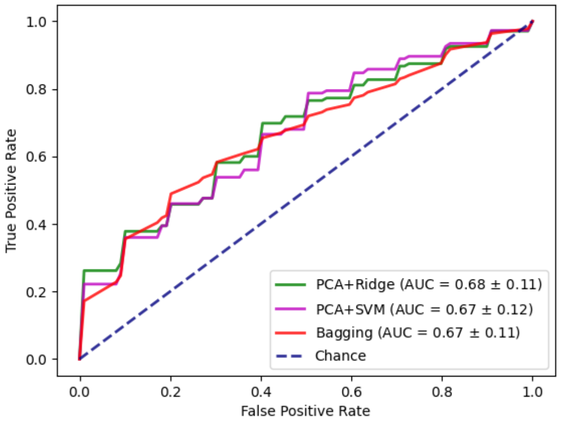

(1j) STG.R

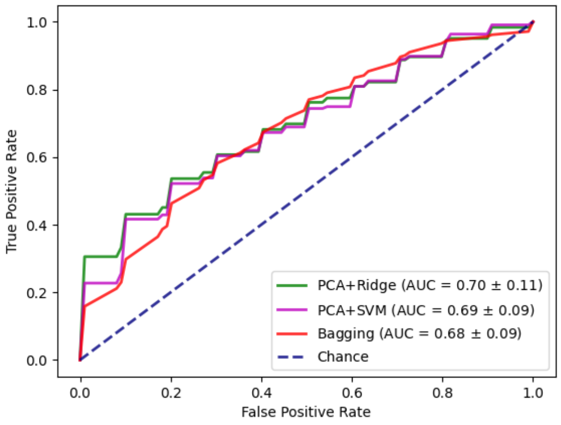

(1k) MTG.L

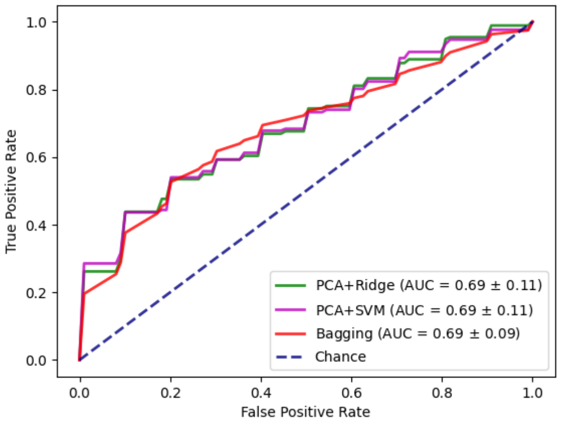

(1l) MTG.R

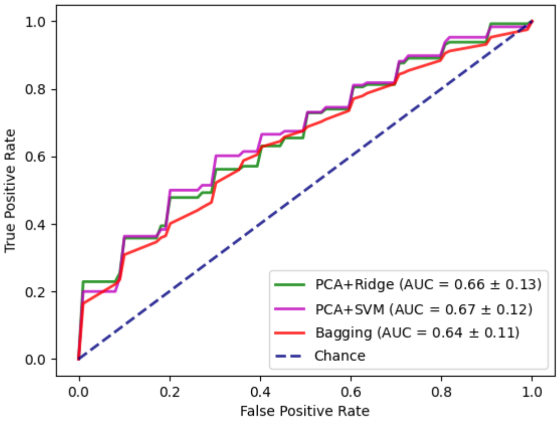

(1m) ITG.L

Figure S1: The ROC curves of different methods on GU for candidate biomarkers including IFGoperc.L(the opercular part of left inferior frontal gyrus),IFGoperc.R(the opercular part of right inferior frontal gyrus),ROL.R(Right Rolandic operculum),OLF.R(right olfactory cortex),REC.R(right Gyrus rectus),INS.R(right insula),IPL.L(left Inferior parietal),STG.R(right temporal gyrus),MTG.L(left middle temporal gyrus),MTG.R(right middle temporal gyrus),ITG.L(left inferior temporal gyrus)

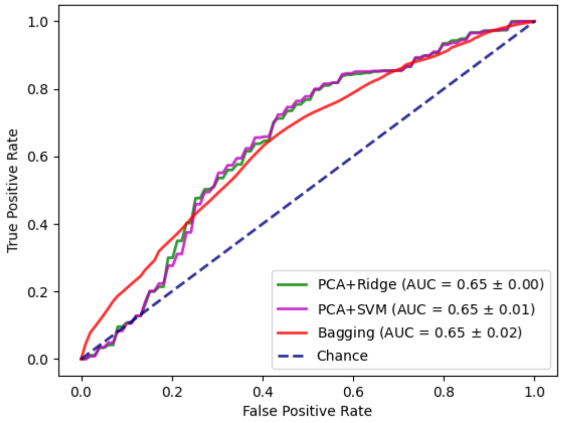

(2n) IFGoperc.L

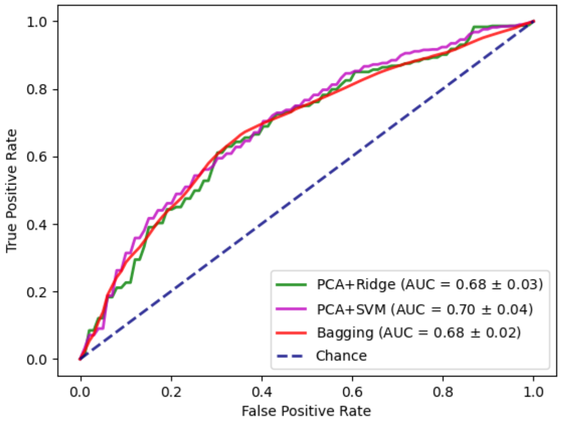

(2o) IFGoperc.R

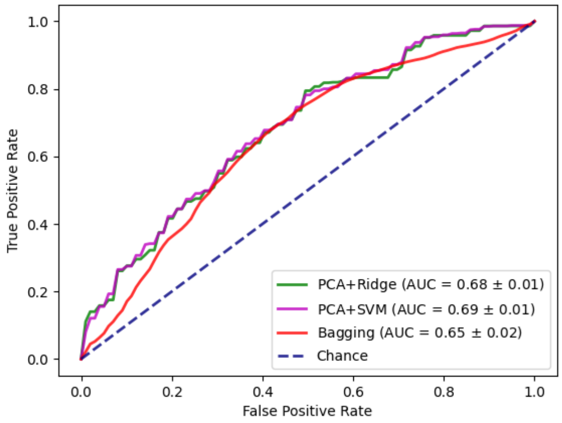

(2p) ROL.R

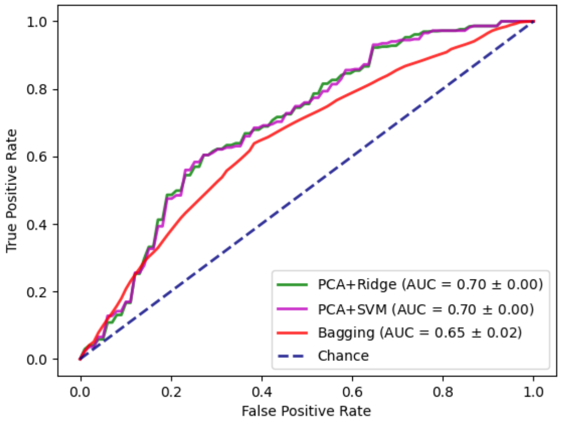

(2q) OLF.R

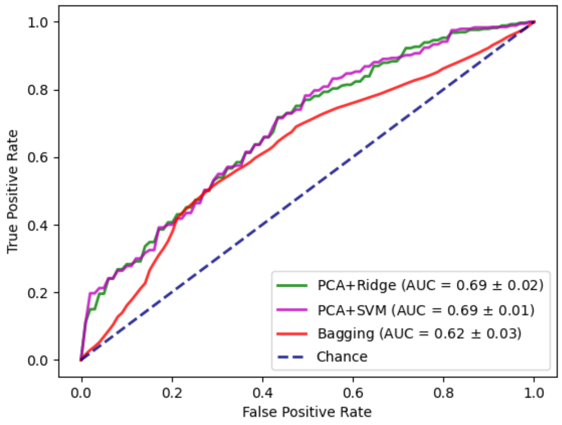

(2r) REC.R

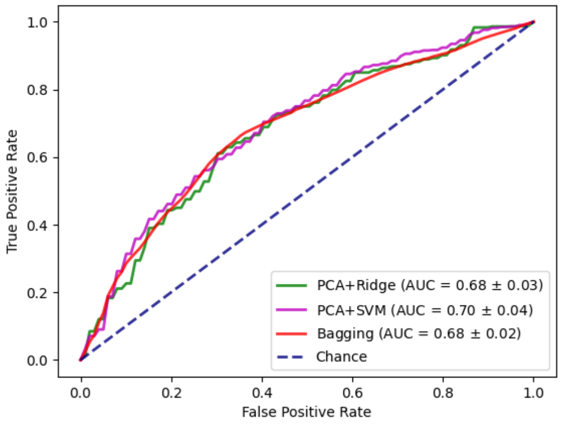

(2s) INS.R

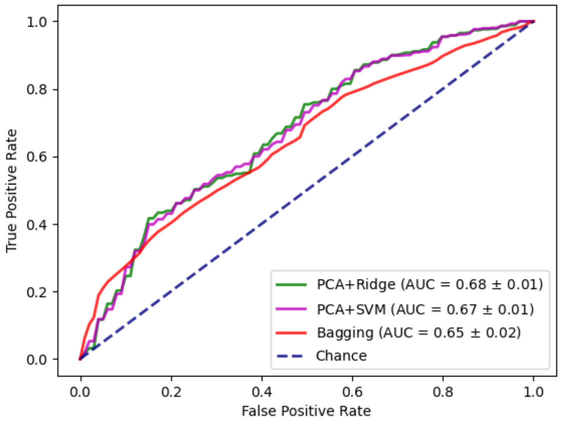

(2t) IFGoperc.L

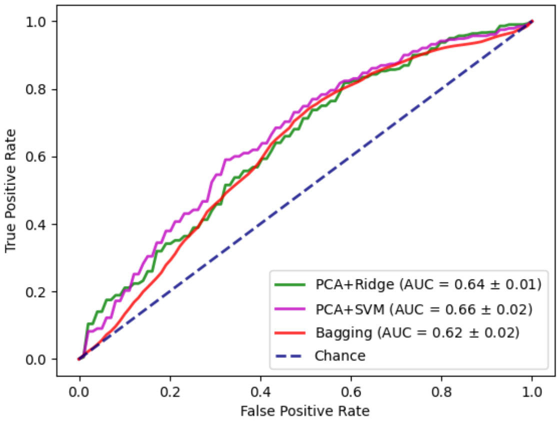

(2u) IFGoperc.R

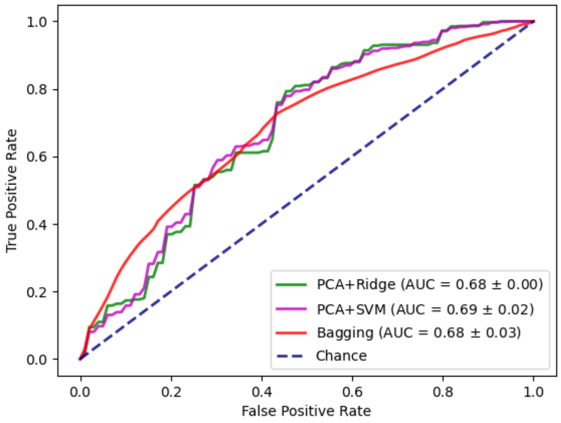

(2v) ROL.R

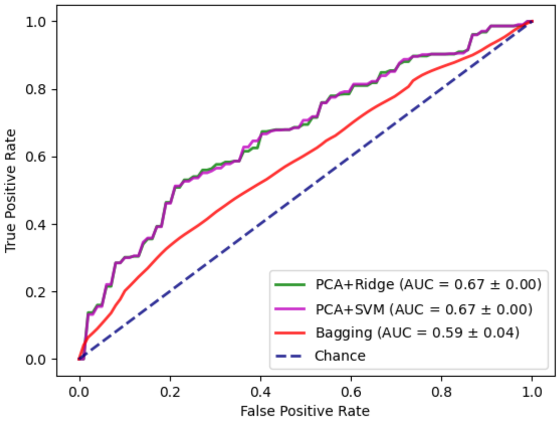

(2w) OLF.R

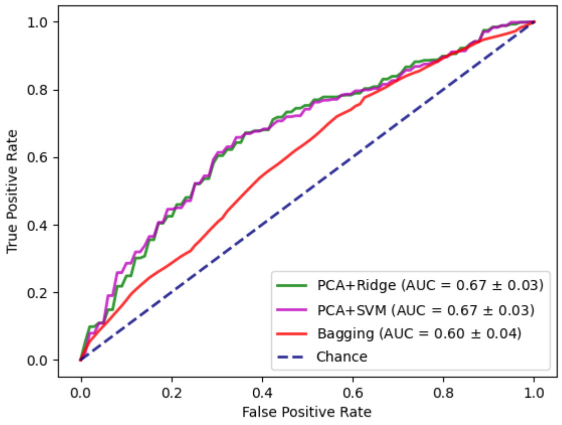

(2x) REC.R

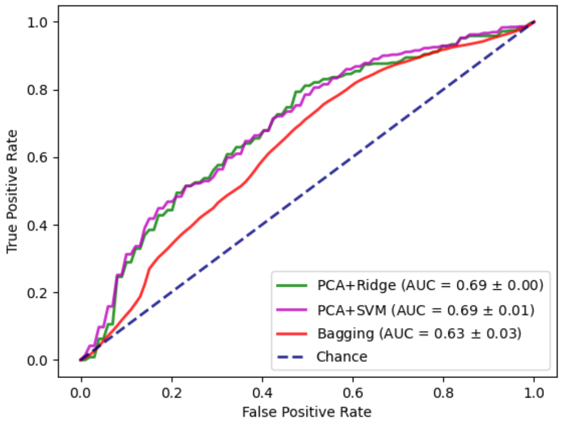

(2y) INS.R

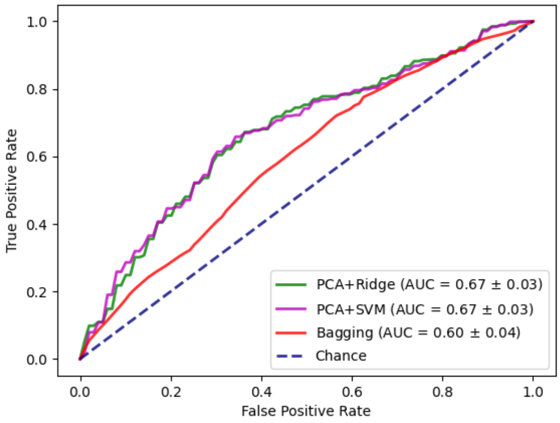

(2z) REC.R

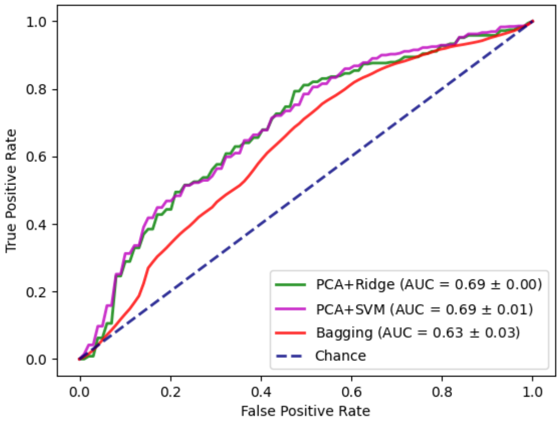

(2) INS.R

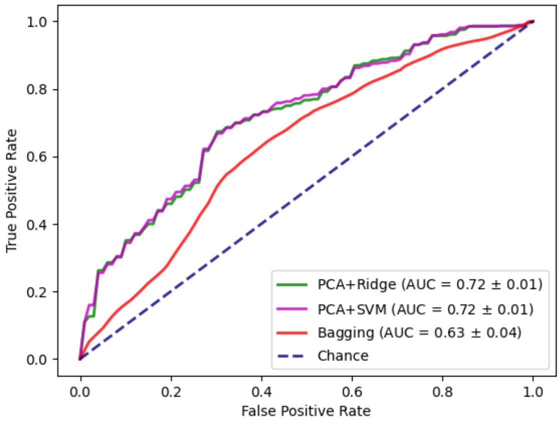

(2) IPL.L

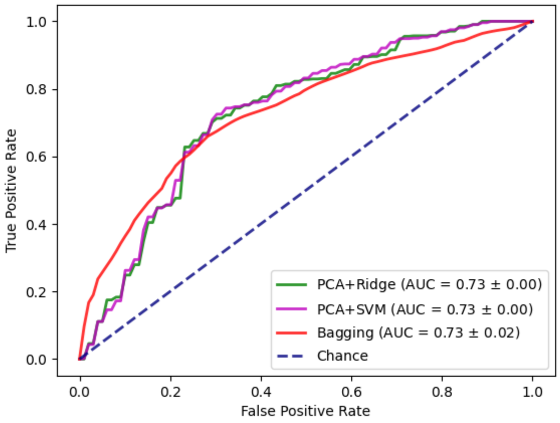

(2) STG.R

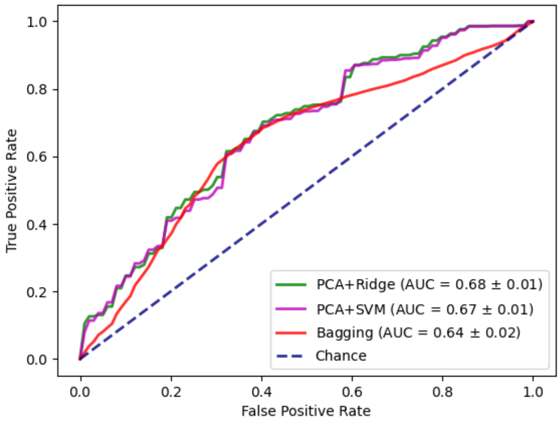

(2) MTG.L

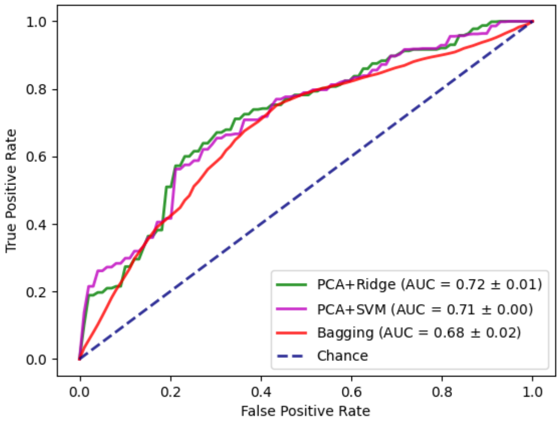

(2) MTG.R

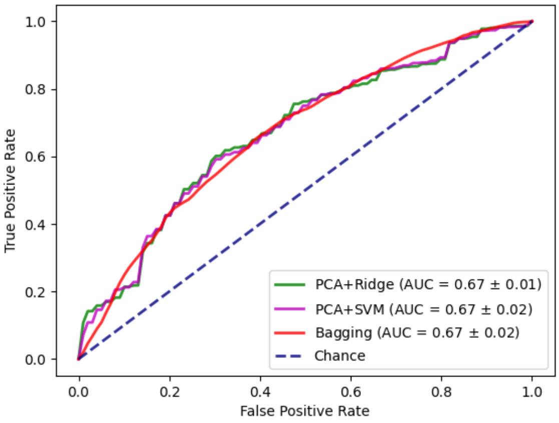

(2) ITG.L

Figure S2: The ROC curves of different methods on multi-site dataset for candidate biomarkers including ORBinf.L(left opercular part of inferior frontal gyrus),STG.L(left superior temporal gyrus),SMG.L(left supramarginal gyrus),SMG.R(right supramarginal gyrus),ANG.L(left angular gyrus),ANG.R(right angular gyrus),IFGoperc.L(the opercular part of left inferior frontal gyrus),IFGoperc.R(the opercular part of right inferior frontal gyrus),ROL.R(Right Rolandic operculum),OLF.R(right olfactory cortex),REC.R(right Gyrus rectus),INS.R(right insula),IPL.L(left Inferior parietal),STG.R(right temporal gyrus),MTG.L(left middle temporal gyrus),MTG.R(right middle temporal gyrus),ITG.L(left inferior temporal gyrus)
